# Supplementary material for: Structural visualization of small molecule recognition by CXCR3 uncovers dual-agonism in the CXCR3-CXCR7 system
Source: Nat Commun. 2025 Mar 28;16:3047. doi: 10.1038/s41467-025-58264-w (PMC11953467; doi:10.1038/s41467-025-58264-w)

Supplementary Information

Structural visualization of small molecule recognition by CXCR3 uncovers dual-agonism in the CXCR3-CXCR7 system

Shirsha Saha[1#], Fumiya K. Sano[2#], Saloni Sharma[1#], Manisankar Ganguly[1], Annu Dalal[1], Sudha Mishra[1], Divyanshu Tiwari[1], Hiroaki Akasaka[2], Takaaki A. Kobayashi[2], Nabarun Roy[1], Nashrah Zaidi[1], Yuzuru Itoh[2], Rob Leurs[3], Ramanuj Banerjee[1*], Wataru Shihoya[2*], Osamu Nureki[2*] and Arun K. Shukla[1*]

[1]Department of Biological Sciences, Indian Institute of Technology Kanpur, Kanpur, India; [2]Department of Biological Sciences, Graduate School of Science, The University of Tokyo, Tokyo, Japan; [3]Amsterdam Institute for Molecules, Medicines, and Systems (AIMMS), Division of Medicinal Chemistry, Faculty of Sciences, VU University Amsterdam, Amsterdam, The Netherlands.

[#]Contributed equally

*Corresponding authors (ramanujb@iitk.ac.in, wtrshh9@gmail.com, nureki@bs.s.u-tokyo.ac.jp, arshukla@iitk.ac.in)

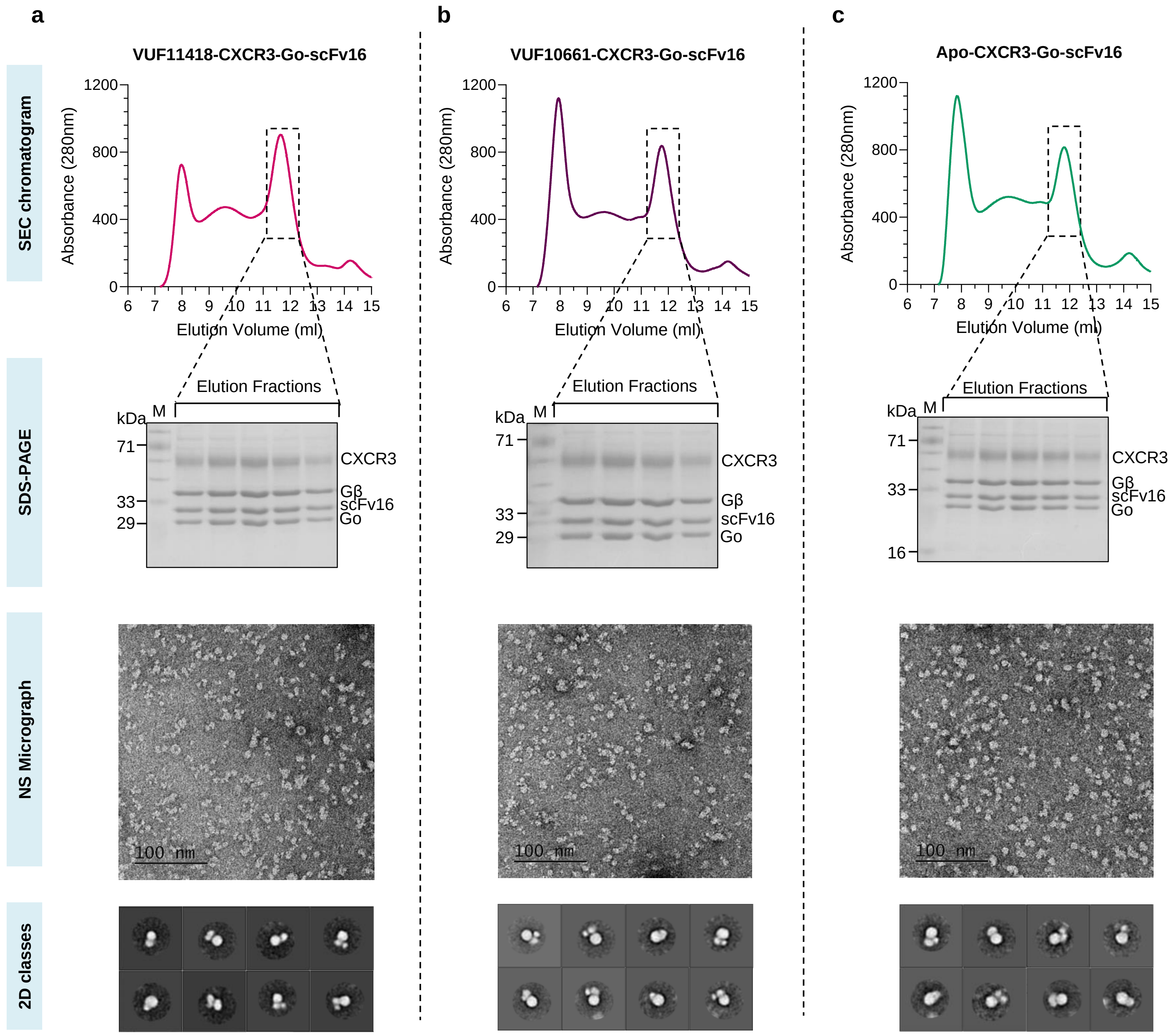

Supplementary Fig. 1: Purification of CXCR3 complexes and visualization through negative-staining EM. a-c, Size exclusion chromatography profile, SDS-PAGE and negative staining-EM of VUF11418-CXCR3-Go-scFv16, VUF10661-CXCR3-Go-scFv16 and Apo-CXCR3-Go-scFv16 complexes, respectively. Source data are provided as a Source Data file.

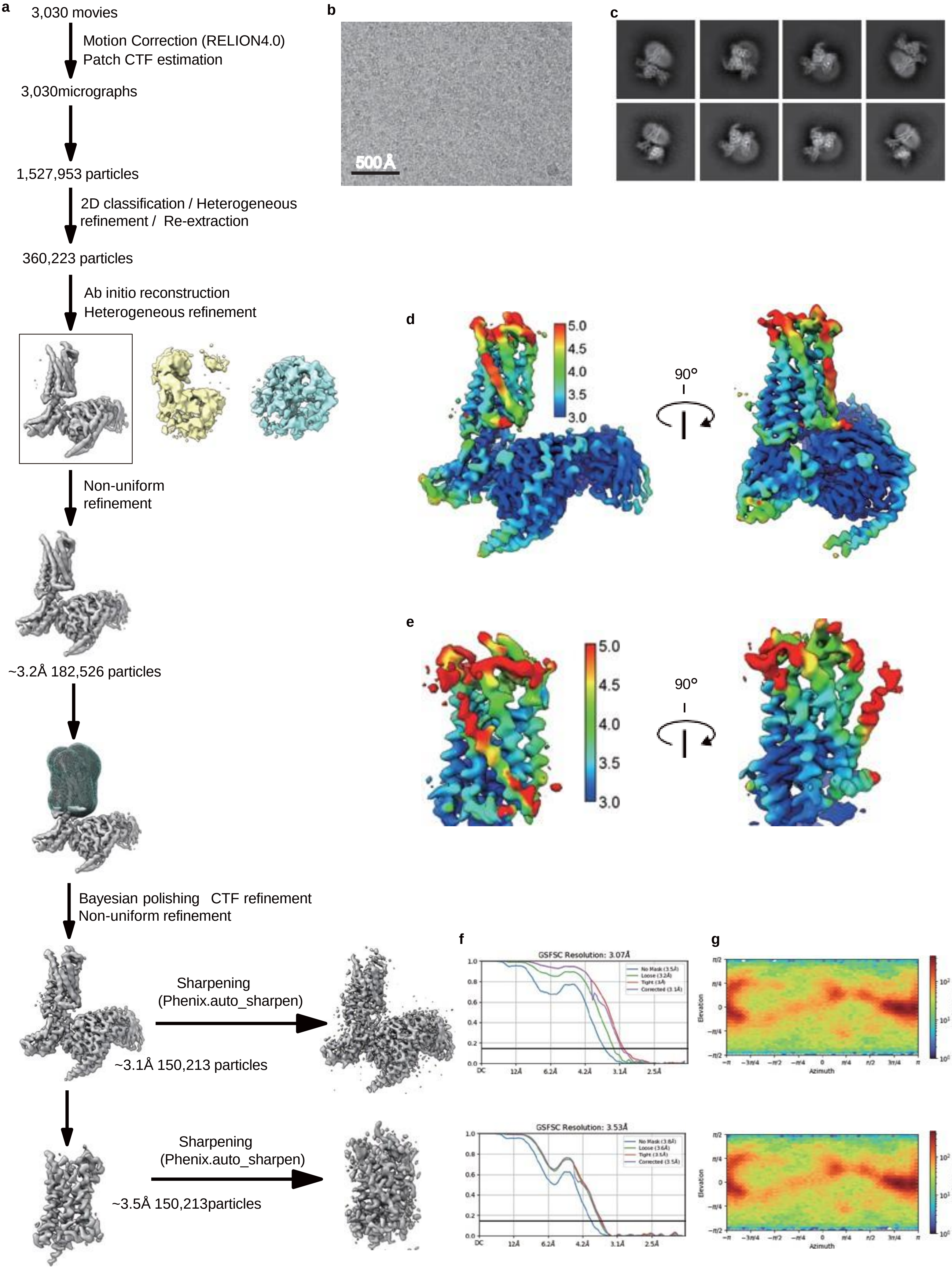
a
3,030 movies
Motion Correction (RELION4.0)
Patch CTF estimation
3,030micrographs
1,527,953 particles
2D classification / Heterogeneous refinement / Re-extraction
360,223 particles
Ab initio reconstruction
Heterogeneous refinement
Non-uniform refinement
~3.2Å 182,526 particles
Bayesian polishing CTF refinement
Non-uniform refinement
Sharpening
(Phenix.auto_sharpen)
~3.1Å 150,213 particles
Sharpening
(Phenix.auto_sharpen)
~3.5Å 150,213particles
b
500 Å
c
d
5.0
4.5
4.0
3.5
3.0
90°
e
5.0
4.5
4.0
3.5
3.0
90°
f
GSFSC Resolution: 3.07Å
GSFSC Resolution: 3.53Å
g
Elevation
Azimuth
# of images

Supplementary Fig. 2: Cryo-EM data processing pipeline of VUF11418-CXCR3-G-protein complex.

a, Schematic representation of the cryo-EM data processing workflow. **b**, A representative cryo-EM image of the VUF11418-CXCR3-G-protein complex, recorded on a 300 kV Titan Krios with a K3 camera. **c**, A representative 2D averages of curated particles. **d-e,** Local resolution of both overall (d) and local (e) refined maps. **f**, Gold standard fourier shell correlation curve (GFSC) at 0.143 threshold for both overall and local refined reconstruction. **g**, Angular distribution of the particles used for final reconstruction.

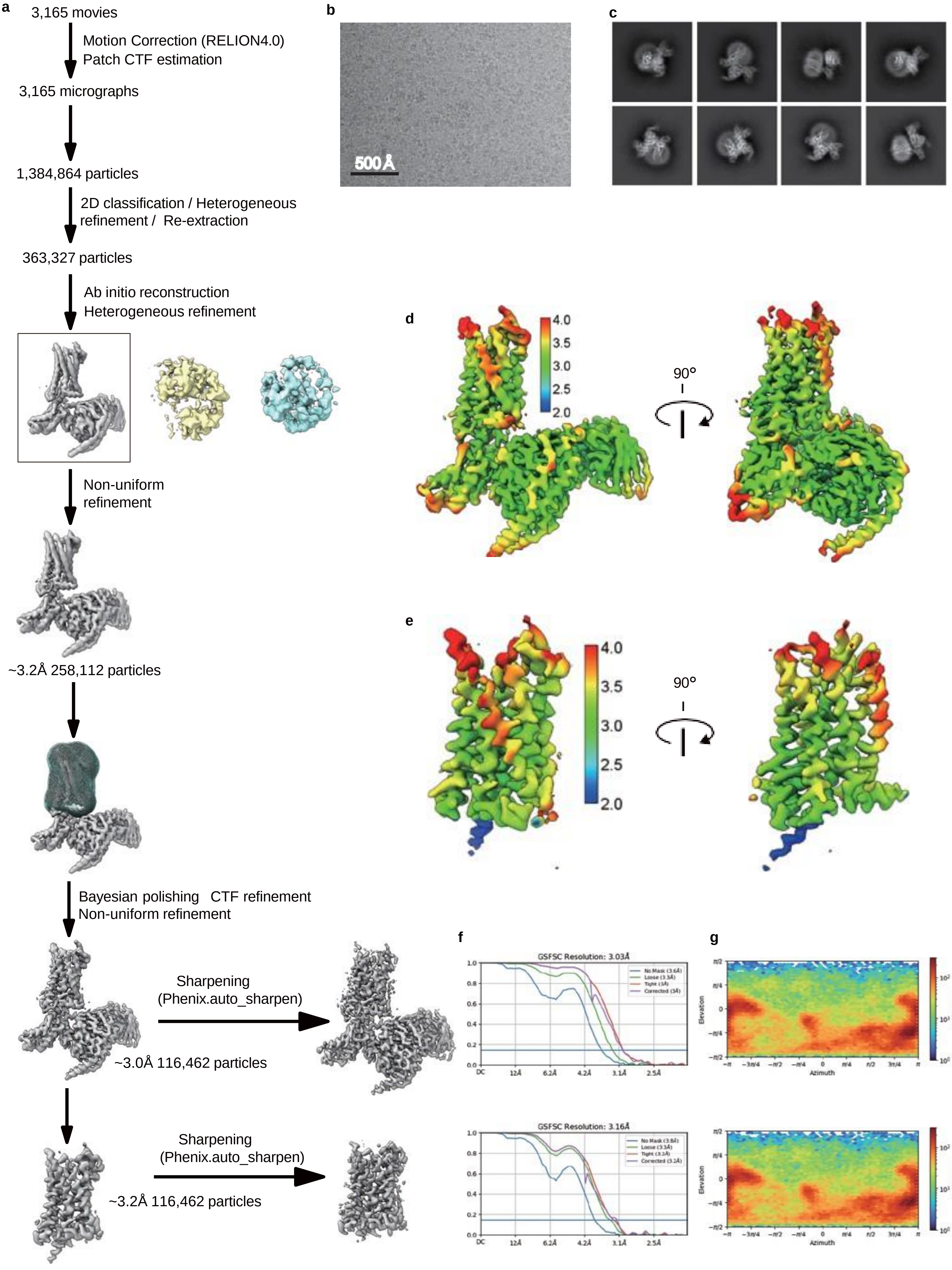

a
3,165 movies
Motion Correction (RELION4.0)
Patch CTF estimation
3,165 micrographs
1,384,864 particles
2D classification / Heterogeneous
refinement / Re-extraction
363,327 particles
Ab initio reconstruction
Heterogeneous refinement
Non-uniform
refinement
~3.2Å 258,112 particles
Bayesian polishing CTF refinement
Non-uniform refinement
Sharpening
(Phenix.auto_sharpen)
~3.0Å 116,462 particles
Sharpening
(Phenix.auto_sharpen)
~3.2Å 116,462 particles
b
500 Å
c
d
4.0
3.5
3.0
2.5
2.0
90°
e
4.0
3.5
3.0
2.5
2.0
90°
f
GSFSC Resolution: 3.03Å
GSFSC Resolution: 3.16Å
g
Elevation
Azimuth
# of images

Supplementary Fig. 3: Cryo-EM data processing pipeline of VUF10661-CXCR3-G-protein complex.

a, Schematic representation of the cryo-EM data processing workflow. **b**, A representative cryo-EM image of the VUF10661-CXCR3-G-protein complex, recorded on a 300 kV Titan Krios with a K3 camera. **c**, A representative 2D averages of curated particles. **d-e,** Local resolution of both overall (d) and local (e) refined maps. **f**, Gold standard fourier shell correlation curve (GFSC) at 0.143 threshold for both overall and local refined reconstruction. **g**, Angular distribution of the particles used for final reconstruction.

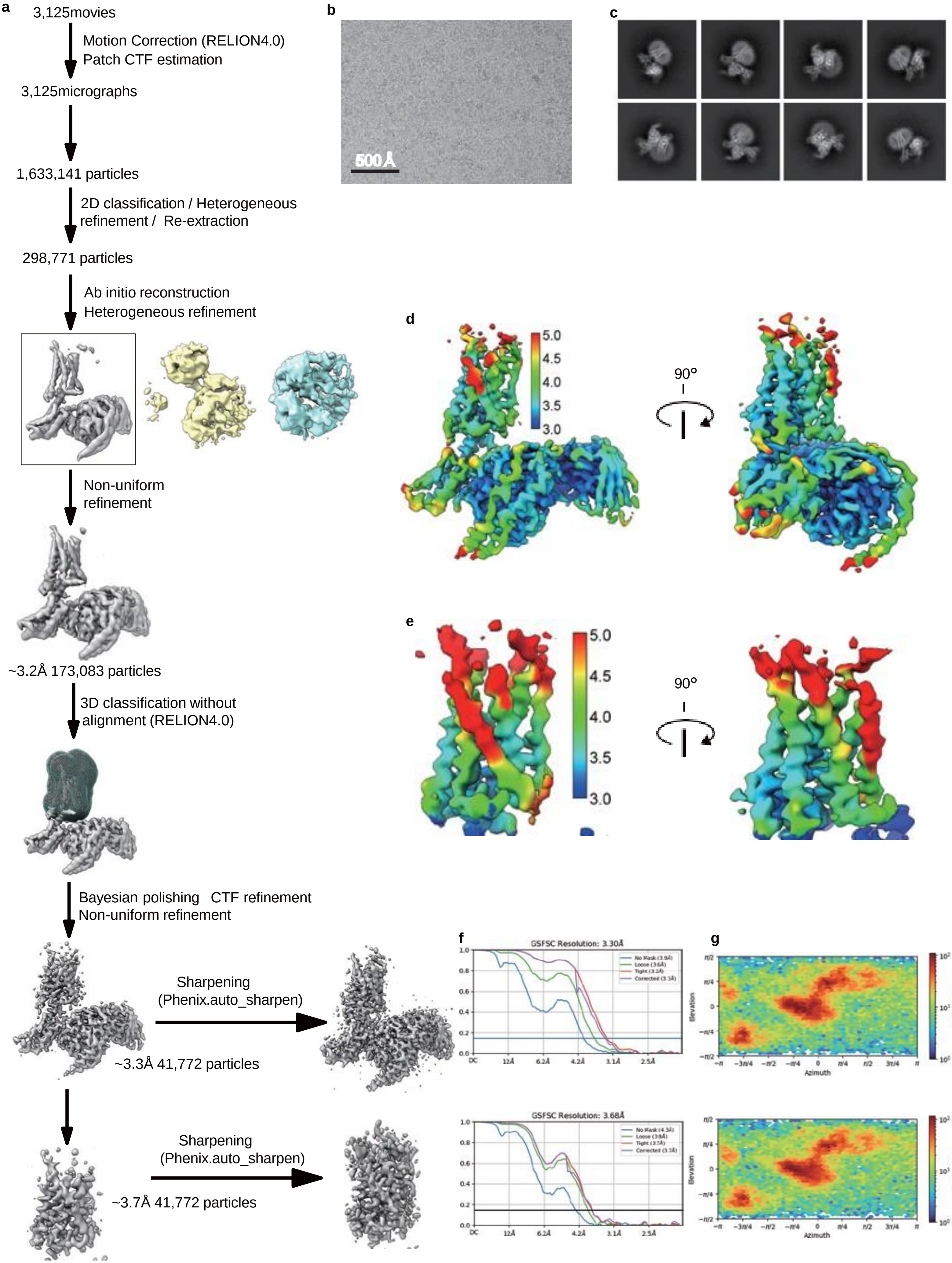
a
3,125movies
Motion Correction (RELION4.0)
Patch CTF estimation
3,125micrographs
1,633,141 particles
2D classification / Heterogeneous refinement / Re-extraction
298,771 particles
Ab initio reconstruction
Heterogeneous refinement
Non-uniform refinement
~3.2Å 173,083 particles
3D classification without alignment (RELION4.0)
Bayesian polishing CTF refinement
Non-uniform refinement
Sharpening
(Phenix.auto_sharpen)
~3.3Å 41,772 particles
Sharpening
(Phenix.auto_sharpen)
~3.7Å 41,772 particles
b
500 Å
c
d
5.0
4.5
4.0
3.5
3.0
90°
e
5.0
4.5
4.0
3.5
3.0
90°
f
GSFSC Resolution: 3.30Å
GSFSC Resolution: 3.68Å
g
Azimuth
Elevation
# of images

Supplementary Fig. 4: Cryo-EM data processing pipeline of Apo-CXCR3-G-protein complex

a, Schematic representation of the cryo-EM data processing workflow. **b**, A representative cryo-EM image of the Apo-CXCR3-G-protein complex, recorded on a 300 kV Titan Krios with a K3 camera. **c**, A representative 2D averages of curated particles. **d-e,** Local resolution of both overall (d) and local (e) refined maps. **f**, Gold standard fourier shell correlation curve (GFSC) at 0.143 threshold for both overall and local refined reconstruction. **g**, Angular distribution of the particles used for final reconstruction.

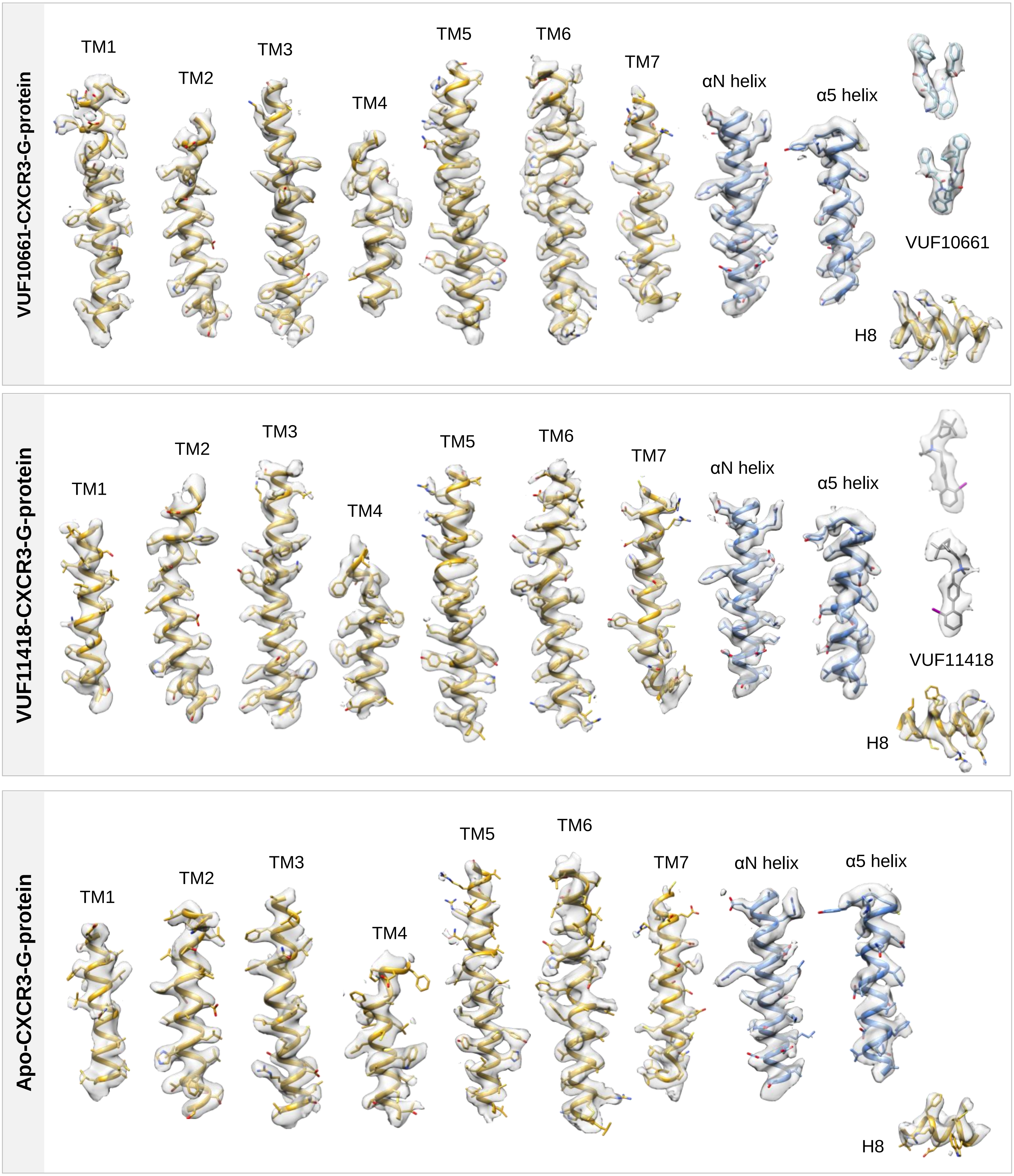

Supplementary Fig. 5: Exemplary electron density maps of the CXCR3-G-protein complexes. EM densities of the TMs and Helix 8 of VUF10661-CXCR3, VUF11418-CXCR3 and Apo-CXCR3, αN helix and α5 helix of miniGαo and VUF10661 and VUF11418.

Subunit	Total residues	Resolved residues	Total residues	Resolved residues	Total residues	Resolved residues
	VUF10661-CXCR3		VUF11418-CXCR3		Apo-CXCR3	
Ligand/ Drug	1	1	1	1	0	0
CXCR3	M1-L368	D46-R335	M1-L368	P57-R335	M1-L368	L59-A110<br>V126-R161<br>P165-I188<br>P208-L332
miniGαo	T4-H57<br>T182-Y354	T4-I56<br>T183-Y354	T4-H57<br>T182-Y354	T4-I56<br>T183-Y354	T4-H57<br>T182-Y354	T4-I56<br>T183-Y354
Gβ	M1-N340	D5-N340	M1-N340	D5-N340	M1-N340	D5-N340
Gγ	M1-L71	I9-F61	M1-L71	I9-F61	M1-L71	I9-F61
scFv16	D1-K248	D1-S121<br>S124-L235	D1-K248	D1-S121<br>S124-L235	D1-K248	D1-S121<br>S124-L235

Supplementary Fig. 6: Residues resolved in the various complexes. Residues resolved in the VUF10661-CXCR3-G-protein, VUF11418-CXCR3-G-protein and Apo-CXCR3-G-protein complexes.

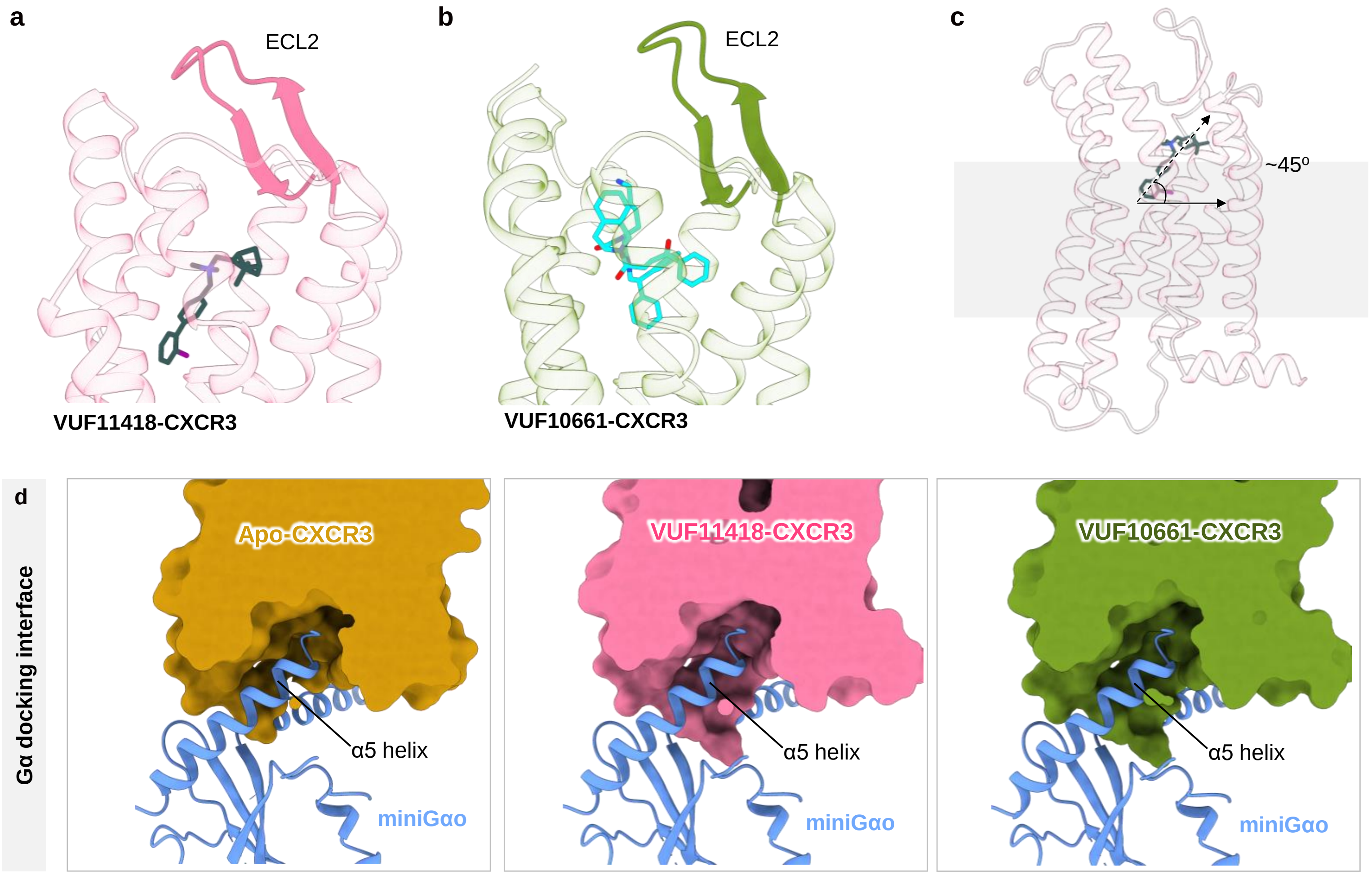

Supplementary Fig. 7: Ligand binding to CXCR3 and G-protein docking interface. a-b, Orientation of VUF11418 and VUF10661 within the orthosteric pocket of CXCR3. ECL2 residues can be seen to adopt a β-hairpin conformation. **c,** Orientation angle of VUF11418 with respect to the lipid bilayer. **d,** Docking of the α5 helix of miniGαo into the cytoplasmic core of CXCR3 in the Apo (left), VUF11418 (middle) and VUF10661 (right) bound CXCR3 structures. (Apo-CXCR3: dark goldenrod, VUF11418-CXCR3: pale violet red, VUF10661-CXCR3: olive drab, Gαo: cornflower blue, VUF11418: deep teal, VUF10661: cyan)

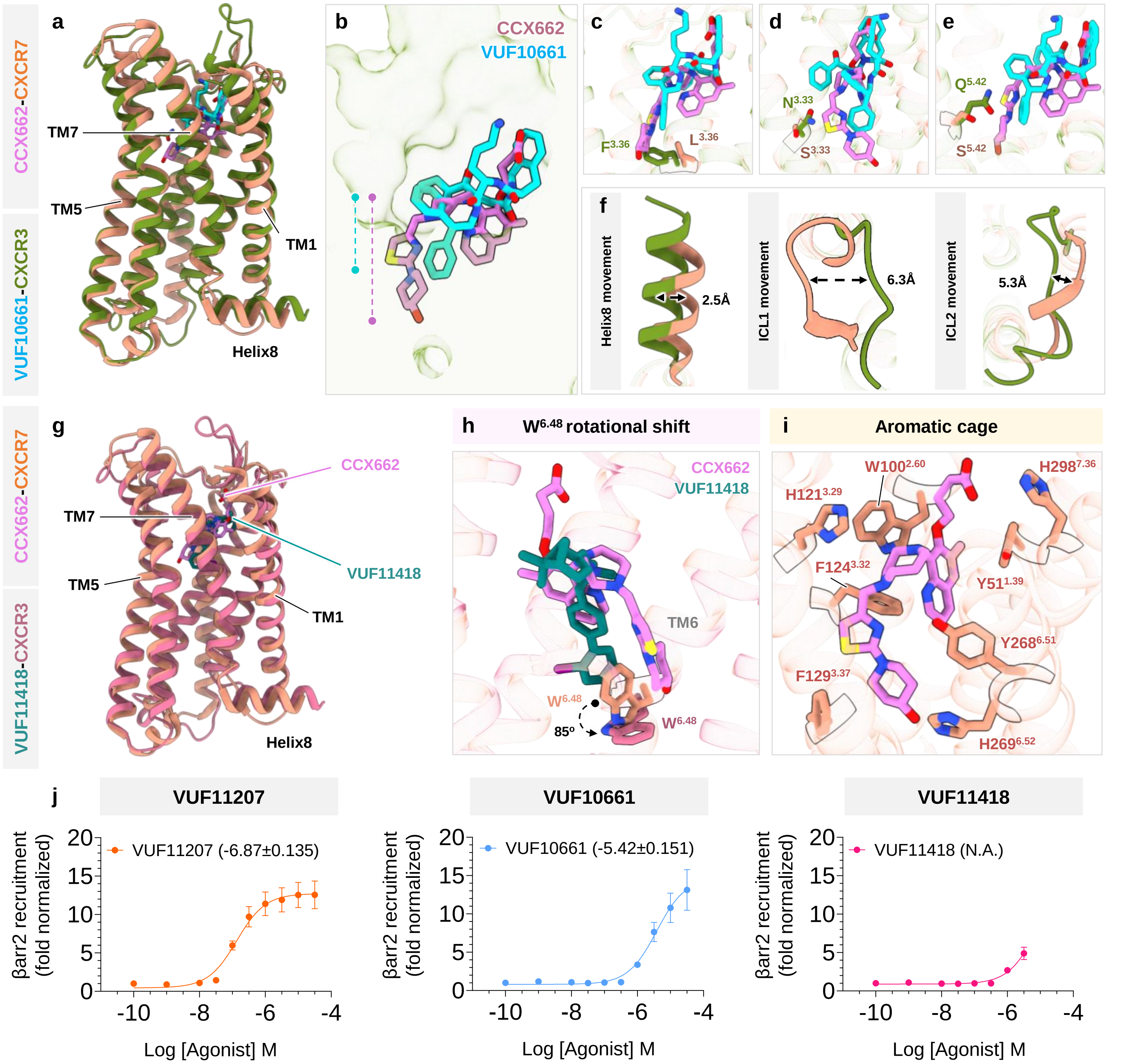

Supplementary Fig. 8: Structural comparison of VUF10661-CXCR3 and VUF11418-CXCR3 with CCX662-CXCR7 and βarr2 recruitment to CXCR7. a, Structural superimposition of VUF10661-CXCR3 (olive drab) with CCX662-CXCR7 (coral). **b**, CCX662 (pink) adopts a different conformation and penetrates deeper into the orthosteric pocket than VUF10661 (cyan), **c-e,** Differential orientation of key ligand interacting residues facilitates differential docking of VUF10661 and CCX662. **f,** Helix8, ICL1 and ICL2 exhibit displacement between VUF10661-bound CXCR3 and CCX662-bound CXCR7. **g,** Structural superimposition of VUF11418-CXCR3 (pale violet red) and CCX662-CXCR7. **h,** $W^{6.48}$ in VUF11418-bound CXCR3 flips away from the ligand binding site. (VUF11418: deep teal) **i,** Aromatic residues surrounding CCX662 in CXCR7. **j,** βarr2 recruitment to CXCR7 as measured by TANGO assay confirms the dual agonistic property of VUF10661 (blue) and VUF11418 (pink). Data (mean±SEM) represents three independent biological replicates, performed in duplicate, and normalized with respect to signal observed at lowest dose, treated as 1. VUF11207 has been used as a positive control for CXCR7 (orange). Source data are provided as a Source Data file.

	Apo-CXCR3-Go (focused)	Apo-CXCR3-Go (overall)	VUF11418-CXCR3-Go (focused)	VUF11418-CXCR3-Go (overall)	VUF10661-CXCR3-Go (focused)	VUF10661-CXCR3-Go (overall)
**PDB ID**	PDB- 8XXY	PDB- 8XXZ	PDB- 8Y0H	PDB- 8Y0N	PDB- 8XYI	PDB- 8XYK
**EMDB ID**	EMD-38765	EMD- 38766	EMD-38803	EMD-38809	EMD-38774	EMD-38776
**Microscope**	Titan Krios	Titan Krios	Titan Krios	Titan Krios	Titan Krios	Titan Krios
**Camera**	GIF/K3	GIF/K3	GIF/K3	GIF/K3	GIF/K3	GIF/K3
**Magnification**	105,000x	105,000x	105,000x	105,000x	105,000x	105,000x
**Voltage (kV)**	300	300	300	300	300	300
**Defocus range (μm)**	-0.8 to -1.6	-0.8 to -1.6	-0.8 to -1.6	-0.8 to -1.6	-0.8 to -1.6	-0.8 to -1.6
**Total dose (e^-/A^2)**	50.1	50.1	50.1	50.1	50.1	50.1
**Pixel size (Å)**	0.82	0.82	0.82	0.82	0.92	0.92
**Micrographs (no.)**	3,125	3,125	3,030	3,030	3165	3165
**Initial particles (no.)**	16,33,141	16,33,141	15,27,953	15,27,953	1,384,864	1,384,864
**Symmetry imposed**	C1	C1	C1	C1	C1	C1
**Final particles (no.)**	44,772	44,772	150,213	150,213	116,462	116,462
**FSC threshold**	0.143	0.143	0.143	0.143	0.143	0.143
**Map resolution (Å)**	3.68	3.3	3.53	3.07	3.16	3.03
**Refinement**						
**Initial model (PDB Code)**	A0A0S2Z3W5	A0A0S2Z3W5, 7XJJ, 7DB6	A0A0S2Z3W5	A0A0S2Z3W5, 7XJJ, 7DB6	A0A0S2Z3W5	A0A0S2Z3W5, 7XJJ, 7DB6
**Model resolution (Å)**	4	3.6	3.7	3.3	3.4	3.2
**FSC threshold**	0.5	0.5	0.5	0.5	0.5	0.5
**Model composition**						
**Non-hydrogen atoms**	1,838	8,331	2,198	8,691	2314	8807
**Protein residues**	237	1074	279	1116	290	1127
**Ligand atoms**	0	0	1	1	1	1
**R.M.S deviation**						
**Bond length (Å)**	0.004	0.005	0.004	0.004	0.003	0.004
**Bond angle (º)**	0.856	0.937	0.911	0.936	0.595	0.911
**Validation**						
**Favored (%)**	99.56	96.3	97.83	97.01	96.88	96.32
**Allowed (%)**	0.44	3.7	2.17	2.99	3.12	3.68
**Disallowed (%)**	0	0	0	0	0	0
**MolProbity score**	1.02	1.4	1.28	1.29	1.18	1.4
**Clash Score**	2.37	3.67	4.69	3.24	2.14	3.6

Supplementary Fig. 9: Cryo-EM data collection and refinement statistics. Data collection and processing parameters corresponding to the structures reported in this study are presented.

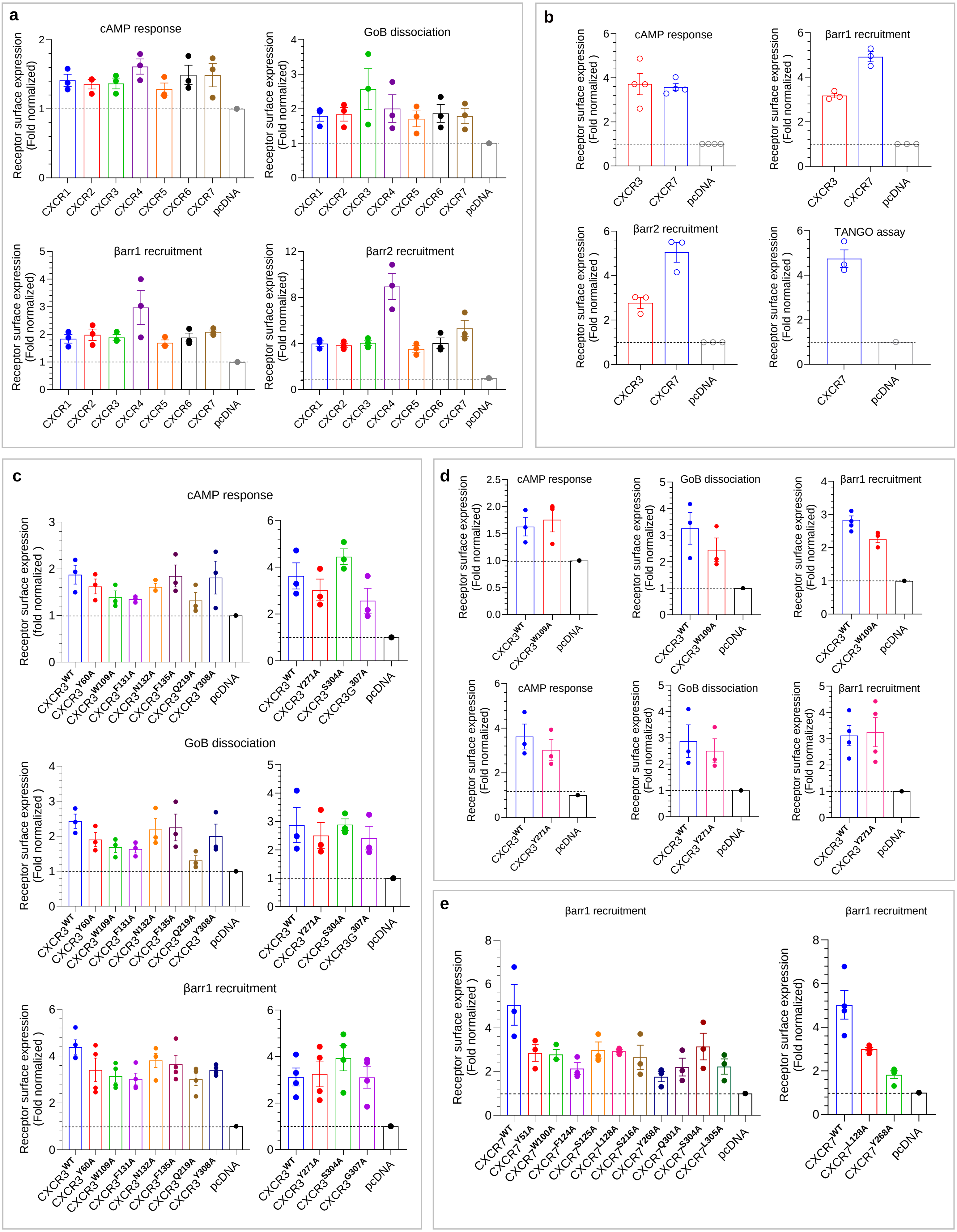
a
cAMP response
GoB dissociation
βarr1 recruitment
βarr2 recruitment
Receptor surface expression (Fold normalized)
CXCR1
CXCR2
CXCR3
CXCR4
CXCR5
CXCR6
CXCR7
pcDNA
b
cAMP response
βarr1 recruitment
βarr2 recruitment
TANGO assay
Receptor surface expression (Fold normalized)
CXCR3
CXCR7
pcDNA
c
cAMP response
GoB dissociation
βarr1 recruitment
Receptor surface expression (fold normalized)
Receptor surface expression (Fold normalized)
CXCR3WT
CXCR3 Y60A
CXCR3W109A
CXCR3F131A
CXCR3N132A
CXCR3F135A
CXCR3Q219A
CXCR3 Y308A
CXCR3 Y271A
CXCR3S304A
CXCR3G307A
pcDNA
d
cAMP response
GoB dissociation
βarr1 recruitment
Receptor surface expression (Fold normalized)
CXCR3WT
CXCR3W109A
CXCR3Y271A
pcDNA
e
βarr1 recruitment
Receptor surface expression (Fold normalized)
CXCR7WT
CXCR7Y51A
CXCR7W100A
CXCR7F124A
CXCR7S125A
CXCR7L128A
CXCR7S216A
CXCR7Y268A
CXCR7Q301A
CXCR7S304A
CXCR7L305A
pcDNA

Supplementary Fig. 10: Surface expression of receptors in various assays.

a, All the receptors used in screening VUF10661 and VUF11418 showed robust expression. **b,** CXCR3 and CXCR7 showed comparable levels of surface expression in the various assays. Surface expression of CXCR7 in TANGO assay is also shown. **c,** All CXCR3 mutants were expressed on the cell surface at levels significantly greater than mock pcDNA transfected cells, as measured using whole-cell surface ELISA. **d,** $CXCR3^{W109A}$ and $CXCR3^{Y271A}$ were expressed on the cell surface at levels comparable to wild type CXCR3. **e,** All CXCR7 mutants were expressed on the cell surface at levels significantly greater than mock pcDNA transfected cells, as measured using whole-cell surface ELISA. Data represents mean±SEM. Source data are provided as a Source Data file.

Supplementary Table 1: List of primers used in this study		
**Construct**	**Primer**	**Sequence**
CXCR1_SmBiT	CXCR1_SmBiT_Fw	CGGGGTACCGAGGAGATCTGCCACCATGGGGAAGACGATCATCGCC
	CXCR1_SmBiT_Rv	TCCCCCGGGCAGGTTGCTGGACACGTTCAC
CXCR2_SmBiT	CXCR2_SmBiT_Fw	CGGGGTACCGAGGAGATCTGCCACCATGGGGAAGACGATCATCGCC
	CXCR2_SmBiT_Rv	TCCCCCGGGCAGGGTGGTGCTGGTGTGGCC
CXCR3_SmBiT	CXCR3_SmBiT_Fw	CGGGGTACCGAGGAGATCTGCCACCATGGGGAAGACGATCATCGCC
	CXCR3_SmBiT_Rv	TCCCCCGGGCAGACCGCTGTAAGAGGCTTCGC
CXCR4_SmBiT	CXCR4_SmBiT_Fw	CGGGGTACCGAGGAGATCTGCCACCATGGGGAAGACGATCATCGCC
	CXCR4_SmBiT_Rv	TCCCCCGGGAGAGCTATGAAATGAGCTGG
CXCR5_SmBiT	CXCR5_SmBiT_Fw	CGGGGTACCGAGGAGATCTGCCACCATGGGGAAGACGATCATCGCC
	CXCR5_SmBiT_Rv	TCCCCCGGGGAAGGTGGTCAGGCTGGTAGCG
CXCR6_SmBiT	CXCR6_SmBiT_Fw	CGGGGTACCGAGGAGATCTGCCACCATGGGGAAGACGATCATCGCC
	CXCR6_SmBiT_Rv	TCCCCCGGGCAGCTGGAACATGGAGGTAGC
CXCR7_SmBiT	CXCR7_SmBiT_Fw	CGGGGTACCGAGGAGATCTGCCACCATGGGC
	CXCR7_SmBiT_Rv	TCCCCCGGGTTTGGTGCTCTGCTCCAAG
CXCR3 (Y60A)	CXCR3 (Y60A)_pcDNA3.1_Fw	GCCTGCTCTGgccAGCCTGCTGTTCCTG
	CXCR3 (Y60A)_pcDNA3.1_Rv	AGGAAAGCGCGGTCGAAG
CXCR3 (W109A)	CXCR3 (W109A)_pcDNA3.1_Fw	CCTGCCTCTGgccGCTGTGGACGC
	CXCR3 (W109A)_pcDNA3.1_Rv	GTCAGCACCAGCAGGGTG
CXCR3 (F131A)	CXCR3(F131A)_pcDNA3.1_Fw	TGGAGCCCTGgccAACATCAACTTCTACGC
	CXCR3(F131A)_pcDNA3.1_Rv	GCCACCTTGCACAGGCCA
CXCR3 (N132A)	CXCR3(N132A)_pcDNA3.1_Fw	AGCCCTGTTCgccATCAACTTCTACGCCG
	CXCR3(N132A)_pcDNA3.1_Rv	CCAGCCACCTTGCACAGG
CXCR3 (F135A)	CXCR3(F135A)_pcDNA3.1_Fw	CAACATCAACgccTACGCCGGTGCTCTGCTG
	CXCR3(F135A)_pcDNA3.1_Rv	AACAGGGCTCCAGCCACC
CXCR3 (Q219A)	CXCR3(Q219A)_pcDNA3.1_Fw	GAGAGTGCTGgccCTGGTGGCCGGTTTCC
	CXCR3(Q219A)_pcDNA3.1_Rv	AGGGCGGTCCTTCCCACC
CXCR3 (Y271A)	CXCR3(Y271A)_pcDNA3.1_Fw	GTGCTGGACCCCCgccCACCTGGTGGTGC
	CXCR3(Y271A)_pcDNA3.1_Rv	CACAGGGCGAAAGCCACC
CXCR3 (S304A)	CXCR3(S304A)_pcDNA3.1_Fw	GAGCGTGACCgccGGTCTGGGCTAC
	CXCR3(S304A)_pcDNA3.1_Rv	TTAGCCACGTCCACGCGA
CXCR3 (G307A)	CXCR3(G307A)_pcDNA3.1_Fw	CTCTGGTCTGgccTACATGCACTG
	CXCR3(G307A)_pcDNA3.1_Rv	GTCACGCTCTTAGCCACG
CXCR3 (Y308A)	CXCR3(Y308A)_pcDNA3.1_Fw	TGGTCTGGGCgccATGCACTGCTG
	CXCR3(Y308A)_pcDNA3.1_Rv	GAGGTCACGCTCTTAGCC
CXCR7 (Y51A)	CXCR7(Y51A)_pcDNA3.1_Fw	CTCCTTCATTgccATTTTCATCTTCGTCATCGG
	CXCR7(Y51A)_pcDNA3.1_Rv	AGCGTGTAGAGCAGGACG
CXCR7 (W100A)	CXCR7(W100A)_pcDNA3.1_Fw	CATCCCAGTCgccGTGGTCAGTCTCG
	CXCR7(W100A)_pcDNA3.1_Rv	GTGAGGACAACCCACAGG
CXCR7 (F124A)	CXCR7(F124A)_pcDNA3.1_Fw	ACACCTCATCgccTCCATCAACCTC
	CXCR7(F124A)_pcDNA3.1_Rv	GTGACTTTGCACGTGAGC
CXCR7 (S125A)	CXCR7(S125A)_pcDNA3.1_Fw	CCTCATCTTCgccATCAACCTCT
	CXCR7(S125A)_pcDNA3.1_Rv	TGTGTGACTTTGCACGTG
CXCR7 (L128A)	CXCR7(L128A)_pcDNA3.1_Fw	CTCCATCAACgccTTCGGCAGCATTTTC
	CXCR7(L128A)_pcDNA3.1_Rv	AAGATGAGGTGTGTGACTTTG
CXCR7 (S216A)	CXCR7(S216A)_pcDNA3.1_Fw	GGAGCTGGTCgccGTTGTCTTGG
	CXCR7(S216A)_pcDNA3.1_Rv	ATGCCGATCAGCCACTCC
CXCR7 (S268A)	CXCR7(Y268A)_pcDNA3.1_Fw	CTGGCTGCCCgccCACGTGGCGG
	CXCR7(Y268A)_pcDNA3.1_Rv	CAGACAAGGAAGACCACCACG
CXCR7 (S301A)	CXCR7(Q301A)_pcDNA3.1_Fw	GCATGTCACAgccTGCCTGTCGCTGGTGCACTGCTG
	CXCR7(Q301A)_pcDNA3.1_Rv	AGGGCCGTGAAGAGGGCG
CXCR7 (S304A)	CXCR7(S304A)_pcDNA3.1_Fw	ACAGTGCCTGgccCTGGTGCACT
	CXCR7(S304A)_pcDNA3.1_Rv	GTGACATGCAGGGCCGTG
CXCR7 (S305A)	CXCR7(L305A)_pcDNA3.1_Fw	GTGCCTGTCGgccGTGCACTGCTGC
	CXCR7(L305A)_pcDNA3.1_Rv	TGTGTGACATGCAGGGCC

Source data: Related to Supplementary Fig. 1a

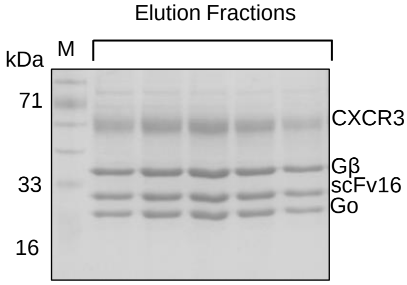

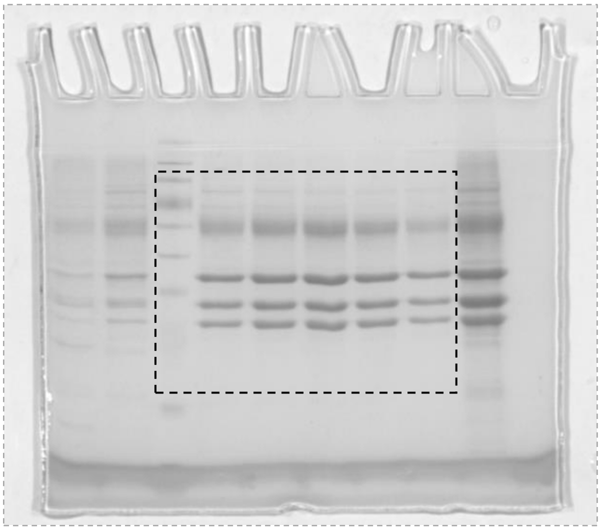

Source data: Related to Supplementary Fig. 1b

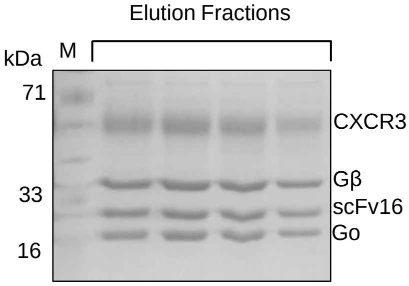

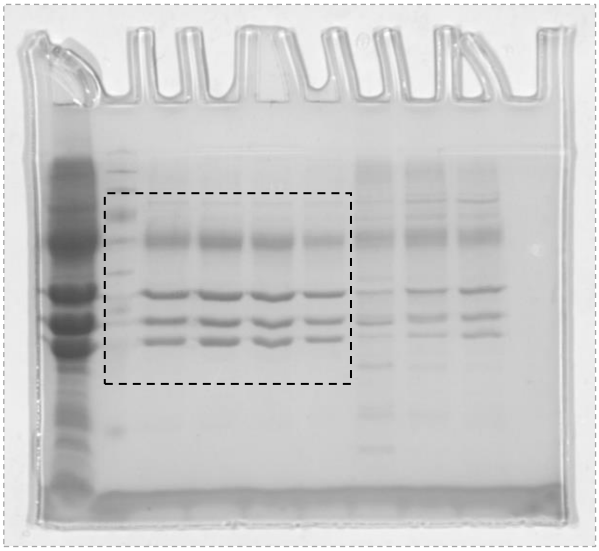

Source data: Related to Supplementary Fig. 1c

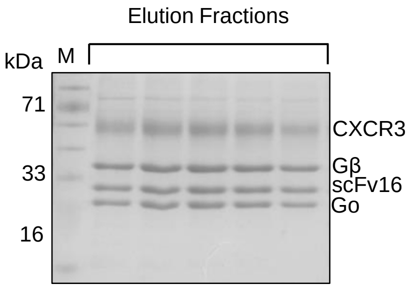

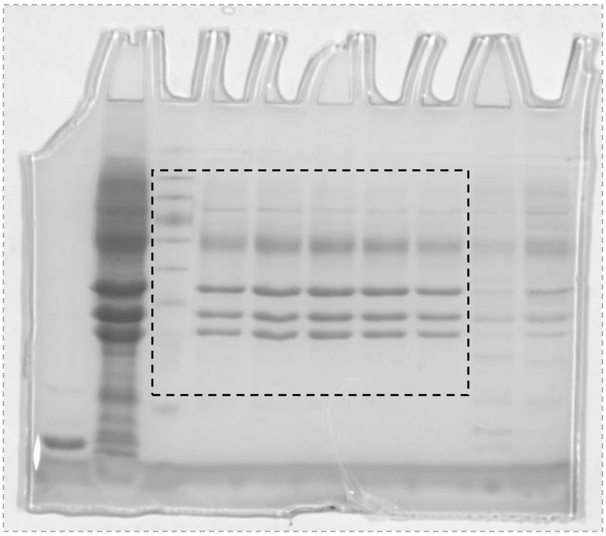

Supplement: Supplementary file 1 — Supplementary Information [file 41467_2025_58264_MOESM1_ESM.pdf]
