# Supplementary material for: Structural visualization of small molecule recognition by CXCR3 uncovers dual-agonism in the CXCR3-CXCR7 system
Source: Nat Commun. 2025 Mar 28;16:3047. doi: 10.1038/s41467-025-58264-w (PMC11953467; doi:10.1038/s41467-025-58264-w)
Supplement: Supplementary file 2 — Description of Additional Supplementary Files [file 41467_2025_58264_MOESM2_ESM.pdf]

Description of Additional Supplementary Files

File name: Supplementary Data 1
Description: List of residue contacts.
Sheet 1: Interaction between VUF10661 and CXCR3.
Sheet 2: Interaction between VUF11418 and CXCR3.
Sheet 3: Interaction between VUF10661 bound CXCR3 and Go.
Sheet 4: Interaction between VUF11418 bound CXCR3 and Go.
Sheet 5: Interaction between Apo-CXCR3 and Go.
